# 

**Published:** 1966-10

**Authors:** 


### BRISTOL MEDICO-CHIRURGICAL JOURNAL

(*Incorporating the Medical Journal of the South-West*)

Established 1 8 8 3

Vol. 81 (iv) October 1966 No. 302

### *Contents*

| | | Page |
|---|---|---|
| THE ADVANCE OF GASTROENTEROLOGY ... | *J. M. Naish* | 58 |
| INTIMATIONS OF SENILITY ........................ | *T. W. Lloyd* | 65 |
| EXPERIENCES IN THE CONTROL OF LONG TERM ANTICOAGULENT TREATMENT ............... | *A. B. Raper* | 68 |
| THE HISTORY OF JAUNDICE IN BRISTOL ... | *Bimla Arora* | 73 |

*PUBLISHED QUARTERLY*

*ANNUAL SUBSCRIPTION* **16/-** *POST FREE*

BRISTOL MEDICO-CHIRURGICAL JOURNAL

PUBLISHED UNDER THE AUSPICES OF THE

### *Bristol Medico-Chirurgical Society*

The Society was founded in 1874. In 1883 publication of this Journal began, and has continued quarterly since then. During the years 1949 to 1962 it appeared under the title of "The Medical Journal of the South West".

The Society founded a medical library in 1890, which in 1892 was combined with that of the Bristol Medical School. This became the University of Bristol Medical Library in 1925, and an agreement in that year confirmed the privilege of Members to use the University Medical Library.

---

### *Editorial Committee*

Dr. N. J. Brown, Southmead Hospital, Bristol. *Joint Editor.*

Dr. A. B. Raper, Department of Pathology, Bristol Royal Infirmary. *Joint Editor.*

Dr. J. Macrae, Ham Green Hospital.

Dr. R. C. Wofinden, Central Clinic, Bristol.

Mr. A. E. S. Roberts, Medical Library, University of Bristol. *Secretary.*

*Local Correspondents*

| | |
|---|---|
| **Bath** . . . | Mr. A. D. Bateman, 3 The Circus, Bath. |
| **Exeter** . . . | Dr. L. N. Jackson, Mount Jocelyn, Crediton, Devon. |
| **Gloucester** . | Dr. R. F. Jarrett, 9 College Green, Gloucester. |
| **Plymouth** . . | Dr. C. R. Croft, Lexden, Hartley Av., Plymouth. |
| **Taunton** . . | Dr. M. McAlley, 1 The Crescent, Taunton. |
| **Torquay** . . | Dr. A. Robinson Thomas, 20 Devon Square, Newton Abbot, Devon. |
| **Truro** . . . | Dr. F. D. M. Hocking, St. Andrews, Perranporth, Cornwall. |
| **Yeovil** . . . | Mr. J. A. Vere Nicoll, Knapp House, Yeovil, Somerset. |

### NOTICE TO CONTRIBUTORS

**Original articles are invited, provided they have not been published elsewhere. Notes of forthcoming events, meetings held, degrees conferred, staff appointments, and other local news are welcomed. The editorial committee reserves the right to make literary corrections.**

**Illustrations should always be supplied ready for reproduction. All re-touchings or other operations required will be charged to the author. Line drawings should be drawn in Indian ink. We regret that we must ask authors to pay for making blocks, if this is necessary.**

### *NEWS AND NOTES*

Mr. D. N. Walder, reader in experimental surgery in the University of Durham, has been appointed to a personal professorship in surgical science.

Professor R. Milnes Walker has been elected a vice-president of the Royal College of Surgeons of England.

Professor T. F. Hewer has been appointed a Pro-Vice-Chancellor of the University of Bristol.

### *Spring Meeting of the Surgical Club of South West England*

The Spring Meeting of the Club was held in Truro on Friday, 6th May 1966. It promised to be a memorable occasion because the Club visited the new hospital at Treliske which was revealed in all its glory in sunshine and sea breezes.

The Meeting will, however, stand out in the minds of those members of the Club who attended as one of the most tragic, because after a most enjoyable day on Friday they learned that one of their hosts, Mr. T. E. Rutter, had died within hours of the end of the dinner. Tim Rutter had read two short and very interesting papers in the morning, and had attended all the sessions during the day. He enjoyed the cocktail party at the Royal Cornwall Yacht Club in Falmouth, and was amongst the number who concluded the day dining at the Royal Duchy Hotel, Falmouth. The Meeting was abandoned on the Saturday, and members set off on their journey home in a sad and reflective mood.

On the Friday, short papers were given as under:

1. "Problems in colonic surgery" — Mr. S. R. Adlington.
2. "Iliocæcal surgery" — Mr. T. M. Reid.
3. (a) "Phlegmasia cerulæ dolens" and
   (b) "Acute inffammation in a solitary cæcal diverticulum"—Mr. T. E. Rutter.
4. "Burns" — Mr. J. R. A. White.

After coffee, Drs. A. K. Thould and J. S. Murrell read a combined paper on "Medical pitfalls in surgical practice" which included malabsorption syndromes, carcinoma of the pancreas, and Cushing's disease.

In the afternoon the Club had a talk from the architect who designed and planned Treliske. This was followed by a tour of the hospital which was a highlight of the Meeting and led to much discussion and a little criticism.

### *Examination Results*

University of Bristol, M.D.: R. J. Ancill.

Ph.D.: R. S. Harris, Rosemary Read.

M.B., Ch.B.: *With Second Class Honours,* Jennifer Bentley, T. G. M. Perham (with Distinction in Public Health).

*Pass*

R. A. S. Balogun

P. A. Boswell

Patricia A. Boulton

Elizabeth E. Bradley

A. R. Colman

April C. Crowther

P. J. Curtis

M. Dunn

D. A. Evans

Maureen M. Franklin

D. R. Gerring

R. V. Hague

J. F. Hamblin

R. A. Hayes

Anne Huggins

A. K. Jethwa

R. Joy

A. C. Knapman

J. D. Minors

Norma Minors

M. G. Mott

C. M. Ndugwa

Carole A. Newman

J. R. Newman

Jane E. Parry

A. J. Pellowe

R. F. Pipe

S. A. F. Quist

A. Resouly

M. R. F. Reynolds

J. A. Richardson

J. M. C. Routh

D. G. Sims

J. T. Siviter

H. G. Smith

P. D. Thompson

Sheila M. Thompson

M. A. Walker

Sally E. Walker

T. A. Wood

D.P.H.:

R. H. Browning

A. J. G. Dickens

R. W. Hollway

Annette M. Lynch (with Distinction)

A. S. Nethercott

M. B. Pepper

A. M. Tucker

